# Autopsied case with MERRF/MELAS overlap syndrome accompanied by stroke‐like episodes localized to the precentral gyrus

**DOI:** 10.1111/neup.12551

**Published:** 2019-04-10

**Authors:** Hiroaki Miyahara, Shinjiro Matsumoto, Kenji Mokuno, Rika Dei, Akio Akagi, Maya Mimuro, Yasushi Iwasaki, Mari Yoshida

**Affiliations:** ^1^ Department of Neuropathology Institute for Medical Science of Aging, Aichi Medical University Nagakute Japan; ^2^ Department of Neurology Toyohashi Municipal Hospital Toyohashi Japan

**Keywords:** frontal cortex, lactic acidosis, MERRF/MELAS overlap syndrome, precentral gyrus, stroke‐like episodes

## Abstract

We present an autopsied case with A8344G‐mutated myoclonus epilepsy with ragged red fibers (MERRF)/mitochondrial encephalomyopathy with lactic acidosis and stroke‐like episodes (MELAS) overlap syndrome accompanied by stroke‐like episodes localized to the precentral gyrus. A 16‐year‐old Japanese woman suddenly experienced repetitive consciousness disturbances with increased serum lactate and creatine kinase levels. Magnetic resonance imaging showed abnormal intensity of bilateral precentral gyrus. She was clinically diagnosed as having a mitochondrial disorder and the A8344G mutation was detected in mitochondrial DNA. At 17 years of age, she died from congestive heart failure secondary to a third episode of lactic acidosis. Neuropatho‐logically, multifocal laminar necrosis, which is responsible for stroke‐like episodes in MELAS, was seen in the frontal cortex including the precentral gyrus, but there was no neuronal loss and gliosis in the basal ganglia, cerebellum, and brainstem, which were compatible with MERRF. Hypertrophy of the vascular smooth muscle and choroidal epithelium were seen, and were strongly visualized by an anti‐mitochondrial antibody. Skeletal muscles showed uneven muscular diameters, increased central nuclei, and ragged red fibers (RRFs). Decreased cytochrome c oxidase (COX) activity and strongly succinate dehydrogenase (SDH)‐reactive blood vessels were also noted. Stroke‐like episodes in MERRF/MELAS overlap syndrome are thought to be rare in the frontal cortex including the precentral gyrus. Only two cases of MERRF/MELAS overlap syndrome with A8344G mutation, including this case, have shown stroke‐like episodes in the frontal lobes. Other than the A8344G mutation and frontal lobe involvement, they had a high degree of similarity in terms of presence of RRFs, gastrointestinal dysfunction, and lack of typical MERRF neuropathology. In conclusion, this is an important case describing the clinical spectrum associated with A8344G‐mutated MERRF/MELAS overlap syndrome.

## INTRODUCTION

We, herein, present an autopsied case with A8344G‐mutated myoclonus epilepsy with ragged red fibers (MERRF)/mitochondrial encephalomyopathy with lactic acidosis and stroke‐like episodes (MELAS) overlap syndrome accompanied by stroke‐like episodes localized to the precentral gyrus.

MERRF (OMIM 545000) and MELAS (OMIM 540000) belong to a heterogeneous group of mitochondrial disorders. Over 80% of MERRF cases involve a heteroplasmic point mutation of A8344G in the mitochondrial tRNA^Lys^ gene,^1^ and are usually accompanied by neurosensory hearing loss, myoclonic epilepsy, and mitochondrial myopathy with ragged red fibers (RRFs). On the other hand, it is believed that the neocortex in patients with MERRF does not show major pathological changes. Patients with MERRF/MELAS overlap syndrome, with the characteristic symptoms of both MERRF and MELAS, have been reported previously,^2^, ^3^, ^4^, ^5^, ^6^, ^7^, ^8^, ^9^, ^10^, ^11^, ^12^, ^13^ but little is known about neuropathological findings in such patients.

We, herein, present an autopsied case with A8344G‐mutated MERRF/MELAS overlap syndrome accompanied by stroke‐like episodes localized to the precentral gyrus.

## CLINICAL SUMMARY

**Figure 1:**
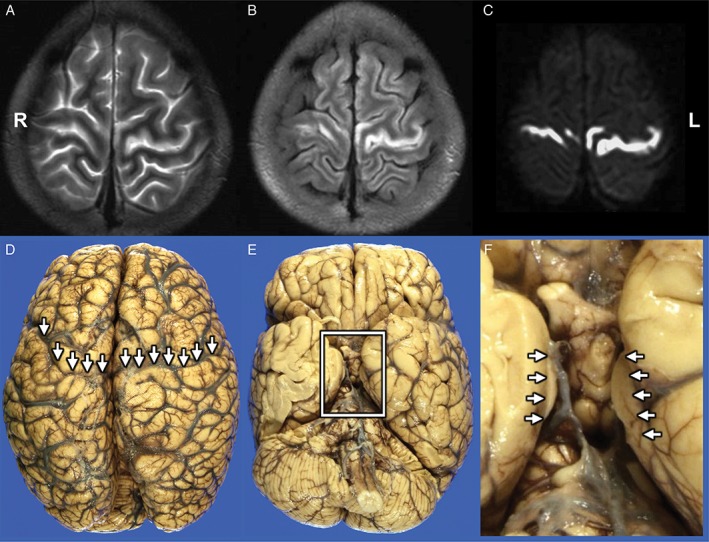
Brain MRI findings (A‐C) and gross appearance of the autopsied brain (D‐F). Hyperintenses areas of both the precentral gyrus are visualized on a T2WI (A), an FLAIR image (B), and a DWI (C). The brain shows massive edema (D, E). Arrows indicate both the precentral gyri (D). Box in (E) and arrows in (F) indicate bilateral uncal herniation.

An autopsy was performed on a 17‐year‐old woman who had a past history of hypertrophic pyloric stenosis in infancy, but not myoclonic epilepsy, neurosensory hearing loss, mental deterioration, or diabetes mellitus. Her mother had a history of myoclonic epilepsy. At 16 years of age, the patient suddenly experienced headache, abdominal pain, nausea, and leg pain, but there was no objective abnormality in her abdominal organs. One month after the onset, she had trouble walking and serum creatine kinase (CK) was elevated. Two months after the onset, she was rushed to the hospital due to generalized convulsions and consciousness disturbance. After admission, generalized convulsions occurred one to two times per day, but there was no prolonged consciousness disturbance. On the fourth day after admission, her consciousness suddenly worsened with lactic acidosis. Her brain MRI showed increased intensity in the bilateral precentral gyrus on T2 weighted images (T2WI), fluid attenuation inversion recovery (FLAIR) images, and diffusion weighted images (DWI) (Fig. 1A–C). She was treated with a vasopressor, antibiotics, and steroid pulse therapy in the intensive care unit, following which she recovered, but the diplegia persisted. She was clinically diagnosed with a kind of mitochondrial disorder on the grounds of lactic acidosis, elevated serum CK, stroke‐like episodes, and family history of myoclonic epilepsy, and an A8344G mutation was confirmed in her mitochondrial DNA. Other mitochondrial DNA mutations including A3243G mutation, which is one of the major gene mutations responsible for MELAS, were not detected. During a third episode of lactic acidosis at the age of 17 years, she died from congestive heart failure. Her autopsy was performed within 70 min after death.

## PATHOLOGICAL FINDINGS

### Macroscopic findings

The whole brain weighed 1130 g before fixation. The gross appearance of her brain showed massive brain edema with cerebrovascular congestion and tonsillar and uncal herniation (Fig. 1D–F).

### Microscopic findings

**Figure 2:**
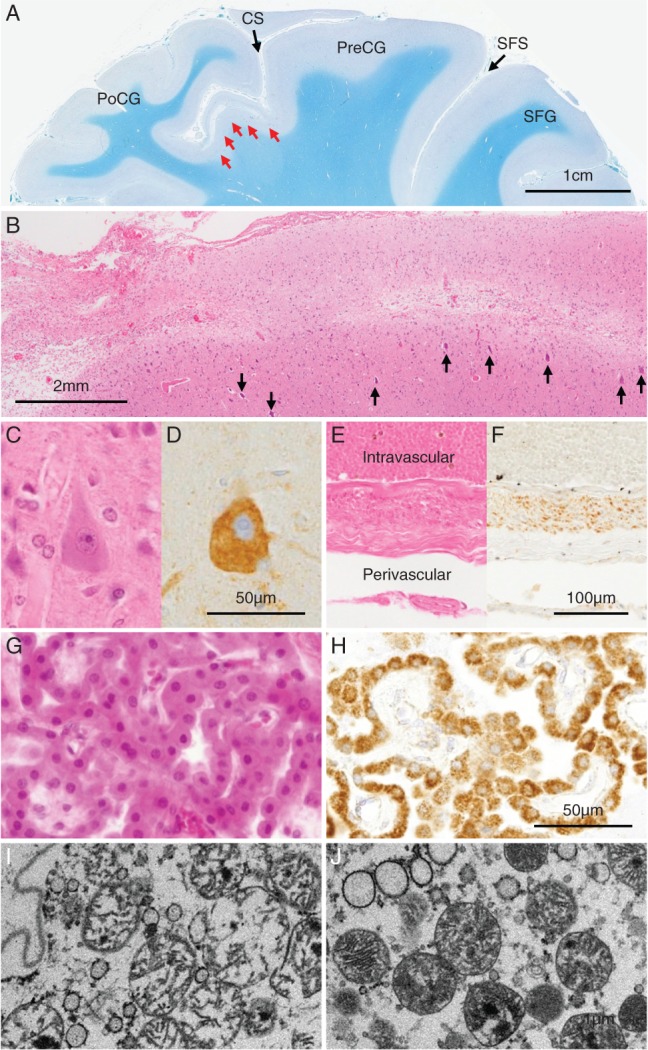
Semimacroscopic (A, B), microscopic (C‐H) and ultrastructural (I, J) findings of the autopsied brain sections stained with KB (A) and HE (B, C, E, G) and immunostained for mitochondria‐specific antigen (D, F, H). (A) A stroke‐like lesion is noted in the precentral gyrus (red arrows). CS: central sulcus; PreCG: precentral gyrus; PoCG: postcentral gyrus; SFS: superior frontal sulcus. (B) A stroke‐like lesion in the precentral gyrus shows laminar necrosis. Betz giant cells (black arrows) are seen below the stroke‐like lesion. (C, D) Unaffected Betz cells look normal, but have abundant mitochondria. (E, F) Vascular smooth muscle cells in the subarachnoid space are hypertrophic and immunoreactive for mitochondria‐specific antigen. (G, H) Choroid plexus epithelial cells are swollen and immunoreactive for mitochondria‐specific antigen. (I) Electron microscopy of a cortical neuron in the present brain reveals damaged mitochondria with irregular and sparse cristae in the choroid plexus epithelial cells. (J) Electrolon microscopy of a cortical neuron in a control brain reveals normal mitochondria. Magnification is the same in (C, D), (E, F), (G, H), and (I, J).

Multifocal laminar necrosis was seen in the precentral gyrus and frontal cortex with pyramidal tract degeneration on sections stained with Klüver‐Barrera (KB) (Fig. 2A). The laminar necrosis closely resembled that caused by stroke‐like episodes of MELAS in microscopic appearance on sections stained with hematoxylin‐eosin (HE) (Fig. 2B). Unaffected Betz giant cells were strongly visualized with an anti‐mitochondrial antibody (Anti‐Human Mitochondria; M117, clone AE‐1, Leinco Technologies Inc., MO, USA), 1:200, using the heat antigen retrieval procedure (98°C for 20 min); they looked normal (Fig. 2C, D). However, there was no neuronal loss and gliosis in the globus pallidus, substantia nigra, cerebellar cortex, spinocerebellar tracts, dorsal columns, Clarke's column, and the red, dentate, and inferior olivary nuclei, which were compatible with MERRF.^14^, ^15^, ^16^

Vascular smooth muscle hypertrophy was often seen in the subarachnoid space (Fig. 2E), and these smooth muscle cells had abundant mitochondria (Fig. 2F). The affected arteries were dilated, and had caused a vasogenic edema. Moreover, gliosis of the thalamic medial nuclei was presumed to be caused by repetetive seizures (data not shown). The vasogenic edema and seizure clusters might have resulted in her massive brain edema. Her choroidal epithelial cells were swollen as previously described in other types of mitochondrial diseases^17^, ^18^, ^19^ (Fig. 2G). Abundant mitochondria in the cytoplasm of the swollen choroidal epithelial cells were labeled by the anti‐mitochondrial antibody (Fig. 2H). Damaged mitochondria with irregular and sparse cristae were seen in the choroidal epithelial cells on electron microscopy (Fig. 2I). Calcium deposition surrounding the vessels and neurons in the cerebral cortex, globus pallidus, and pontine base were also noted (data not shown).

**Figure 3:**
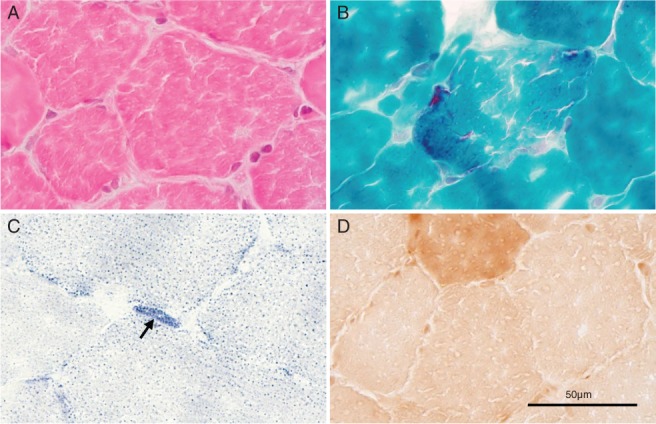
Microphotographs of patient's iliopsoas muscle biopsy specimen sections processed for staining with HE (A) and GT (B) as well as enzyme histochemistry for SDH (C) and COX (D). The muscle tissue shows uneven diameter of muscle fibers (A). RRFs appear as focal aggregates and are observed in the affected muscle fibers (B). Strongly SDH‐reactive blood vessels are observed (C, arrow). Muscle fibers with significantly decresed COX reactivity are observed.

Her iliopsoas muscles showed uneven muscular diameters and increased central nuclei, and reconstruction images demonstrated RRFs (Fig. 3A). Gomori‐trichrome (GT) staining highlighted numerous RRFs in the affected muscles (Fig. 3B). Succinate dehydrogenase (SDH) staining showed strongly SDH‐reactive blood vessels (SSVs) in her intramuscular blood vessels (Fig. 3C). In addition, cytochrome c oxidase (COX) activity was significantly decreased in the affected muscle fibers and blood vessels (Fig. 3D). The above findings were compatible with MERRF but not with MELAS.

## DISCUSSION

Our 17‐year‐old patient with RRFs and A8344G mutation of a mitochondrial gene clinically manifested with repetitive lactic acidosis and stroke‐like episodes of the precentral gyrus, but lacked typical MERRF symptoms such as myoclonus epilepsy and ataxia. Neuropathologically, multifocal laminar necrosis was seen in the precentral gyrus and frontal cortex, but neuronal loss and gliosis in the dentate, red, and inferior olivary nuclei, which are the most typical neuropathological features of MERRF,^14^, ^15^, ^16^ were absent.

As shown in Table 1, in the 12 reviewed cases with MERRF/MELAS overlap syndrome, stroke‐like episodes of MERRF/MELAS overlap syndrome were seen predominantly in the occipital, parietal, and temporal lobes, but rarely in the frontal lobes, including the precentral gyrus. Only one woman had stroke‐like episodes in the frontal lobe, and she had a high degree of similarity with our patient in terms of A8344G mutation, RRFs with deficient COX activity, stroke‐like episodes in the frontal lobes, gastrointestinal dysfunction, short disease duration, and lack of typical MERRF neuropathology. It is difficult to determine whether the co‐occurrence of frontal lobe involvement and A8344G mutation was incidental or a relevant fact, because few cases have been reported previously. Since Betz giant cells in the precentral gyrus commonly require an enormous amount of energy provided by numerous mitochondria, mitochondrial dysfunction is thought to be a possible cause of precentral gyrus involvement. Moreover, the reason for the two cases of MERRF/MELAS overlap syndrome with A8344G mutation not showing typical MERRF lesions was thought to be the short disease duration, which precludes the formation of the lesions to the degree seen in typical MERRF patients.

**Table 1:** Review of 12 cases with MERRF/MELAS overlap syndrome

#	Sex	Age (years)	Duration (year)	Mutation of mt.DNA	Ataxia	Deaf‐ness	ME	RRFs	COX	SSVs	Lactic acidosis	Stroke‐like episodes	GID	References
1	F	17	1	A8344G	−	+	−	+	−	+	+	F (M)	+	This case
2	F	39	3	A8344G	−	+	−	+	−	+	+	O, F	+	12
3	F	39	5	A3243G	−	+	−	+	−	+	+	T		11
4	F	23	6	T8356C + A3243G	N/A	+	+	+	−	+	+ (CSF)	−		9
5	F	46	20	T8356C + A3243G	+	+	+	+	−	+	+	O, P, T		9
6	F	21	3	T8356C + A3243G	+	−	+	N/A	N/A	N/A	+	−		9
7	M	13	5	8356	+	+	+	+	−	+	+	T, P, O		10
8	F	55	42	8356	+	+	−	−	−	−	−	O		10
9	F	31	20	8356	+	+	+	N/A	N/A	N/A	N/A	P		10
10	F	19	11	8356	+	+	+	−	+/−	+	+	−		10
11	F	19	5	T3291C	+	N/A	+	+	−	+	+	O, P, T	+	5
12	M	14	12	3243	+	−	+	+	−	N/A	+	O		2
13	M	25	8	G13042A (ND5)	−	−	+	−	N/A	+	−	P, IC		7

COX, cytochrome c oxidase; F, female; F, frontal lobe; GID, gastrointestinal dysfunction; IC, internal capsule; M, male; M, precentral gyrus; ME, myoclonic epilepsy; N/A, not applicable; O, occipital lobe; P, parietal lobe; RRFs, ragged red fibers; SSVs, strongly Succinate dehydrogenase reactive blood vessels; T, temporal lobe.

Swelling of choroid plexus epithelial cells has been reported in Kearns‐Sayre syndrome,^19^ Leigh encephalopathy,^18^ and MELAS,^17^ but not in MERRF. Such cellular swelling is considered the morphological expression of a biochemical defect in the mitochondrial metabolism, and is associated with increased CSF levels of lactate and pyruvate in these diseases. Since our MERRF case also had choroidal epithelial cell swelling with abundant mitochondria, this might be a common finding in mitochondrial disease.

Further reports should be accumulated to clarify the relationship between A8344G mutation and precentral gyrus involvement in patients with MERRF/MELAS overlap syndrome; nevertheless, this is an important report describing the clinical spectrum associated with A8344G‐mutated MERRF/MELAS overlap syndrome.

## DISCLOSURE

The authors declare that there are no conflicts of interest.
